# Abstract of a Paper Read before the Hunterian Society

**Published:** 1833-04

**Authors:** William Coulson

**Affiliations:** Surgeon to the General Dispensary, and Consulting Surgeon to the London Lying-in Hospital.


### SWELLING OF THE EXTREMITIES AFTER DELIVERY.

*Abstract of a Paper read before the Hunterian Society, by* WILLIAM COULSON, Esq., Surgeon to the General Dispensary, and Consulting Surgeon to the London Lying-in Hospital.

### CASE I. *Swelling of the Right Leg and Left Arm after Parturition; Death.*

SARAH REYNOLDS, aged twenty years, of a delicate habit and fair complexion, was seized, on Thursday, the 22d of October, (fourteen days after delivery,) with headach and numbness of the right ankle. On the following day (the 23d), she was greatly distressed *with pain and swelling of the right leg and left arm;* there was a blush of redness at the ankle and at the wrist; pulse small, quick, and fluttering, (144 in a minute;) skin hot and dry; tongue parched, and rather brown; alvine and urinary secretions natural; continued to suckle her infant; lochia not suppressed.

On the 24th, the symptoms continued nearly the same; but, on the 25th, she began to sink, and expired early in the morning of the 26th.

The body was examined thirty hours after death. The spermatic veins of both sides were carefully traced, and found quite healthy; the veins of the pelvis, with the exception of the right external iliac, were also in a natural state: this vein was a little thicker and more vascular than natural, and contained some coagulable lymph. The uterus was not diseased, nor indeed could any disease be ascertained in the abdomen. The femoral vein was quite healthy. There was a considerable effusion of sero-purulent matter beneath the skin covering the right ankle-joint, and a small quantity beneath the integuments covering the wrist. Permission could not be obtained to examine the other parts of the body.

CASE II. *Swelling of the Upper Extremities after Delivery; Death.*

Sarah Milne, aged eighteen, was seized, at six P.M., December 26th, with shiverings; these were followed by aching pains over the whole body, particularly in the joints; great heat and dryness of the skin, and also occasional delirium. The pulse was extremely quick (140); tongue white and clammy; face pale and countenance anxious; bowels costive.

Some aperient medicines were ordered; and, in case the delirium continued, twelve leeches were directed to be put to the temples.

December 27th. Has passed a restless night, the delirium continuing at intervals. The leeches were applied, and bled well. The lochial discharge continues. The infant died in convulsions this morning, and at present there is no secretion of milk.

One P.M. *The upper extremities are swollen*, from near the axilla to the wrist-joints, and over the *left wrist there are two or three faint red spots*. Pressure on any part of the limb gives great pain, particularly in the course of the vessels and on the joints. The swelling is very tense, but does not pit on pressure. The knee and ankle-joints are very painful, but the lower limbs are not swollen. The patient complains of great thirst; the skin is hot and dry; the action of the pulse rather strong, and very rapid; slight delirium, and a tremulous convulsive action of the muscles of the face.—V. S. ad ℥xij. Saline mixture, with diaphoretics.

The patient felt easier after the bleeding, but died at six P. M.

The body was examined on the following day. There was no organic disease in any part, but there was considerable effusion into the subserous cellular tissue of the abdomen; also between the pia mater and arachnoid; and the pericardium contained above an ounce more of fluid than natural. The swelling of the upper extremities was occasioned by effusion of serum into the cellular tissue. The sinuses of the brain, as well as the veins of the abdomen, pelvis, and upper extremities, were carefully examined, but presented no traces whatever of disease.

CASE III. *Swelling of the Right Knee-joint after Parturition; Death.*

Jane House, aged thirty-two, of a delicate constitution, was seized, fourteen days after delivery of her fourth child, with *severe pain of the right knee-joint:* in a short *time the knee began to swell, and a blush of redness* made its appearance on the integument covering the joints. The pain was aggravated on pressure or motion of the part. The constitutional symptoms had very much the same character in this as in the preceding cases, but the local affection was much severer, the pain and swelling of the joint being greater. This case terminated fatally in six days.

The body was examined twenty-four hours after death, There was a considerable effusion of turbid serous fluid into the abdomen, and the peritoneum covering the pelvis and abdominal viscera was inflamed. The uterus itself was a little softer than natural, but no trace of disease could be detected in the veins of the pelvis or in the right femoral vein. There was a considerable effusion of serum into the cellular tissue covering the right knee-joint.

Two other cases of this kind have occurred in the Lying-in Hospital this year: one terminated fatally in three weeks, but no examination was allowed to be made of the body; in the other, the patient was removed from the hospital during the progress of the complaint, and I am unacquainted with the result of the case.

Although so much attention has of late been paid to diseases of the joints in particular, as well as to the various affections which occur in the puerperal state, little or no notice has been taken of that peculiar affection which forms the characteristic feature of the cases that have just been related. Dr. DENMAN, in his Introduction to Midwifery, p. 570, makes the following observations, which I presume had reference to this form of disease. "There is a peculiarity," says Dr. D. "in this (puerperal) fever, which I believe has not hitherto been observed or mentioned. It is an erysipelatous tumor, of a dusky red colour, on the knuckles, wrists, elbows, knees, or ankles, about the size of a shilling, and sometimes larger. This is almost universally a mortal sign, and, on the inspection of those who have died with this appearance, the disease has been found to have affected principally the uterus or its appendages."

The patient is first attacked with shiverings and aching pains in the limbs, or in one or more joints of the body. The painful joint soon begins to swell, and at the same time a red spot makes its appearance. Sometimes, however, the swelling is not confined to the joint, but extends along the whole of the limb, and in this case faint red spots are generally seen here and there in the course of the swelling. Sometimes also a circumscribed swelling will occur above the joint, neither the joint nor any other part of the limb being affected. If the patient is not rapidly cut off, several joints in succession are attacked in this manner; those first affected sometimes becoming better, at others remaining just as they were. In the last case which occurred in the hospital, the right knee-joint was first affected, but at the end of a fortnight, just when the complaint appeared mitigated, the left ankle-joint was attacked by the same disease. By whatever name we distinguish the fever which accompanies this disease, it is essential to be aware that no one set of internal organs is uniformly affected; sometimes the head is the only part, at other times the chest or abdomen; so that Dr. Denman's assertion, that the uterus and its appendages are principally affected, has not been corroborated by the appearances found in the dissection of the cases which have come under my own observation. The fever is of a subinflammatory kind; the pulse at the commencement strong, but soon becomes quick and small, the tongue is white or of a slate-colour, and, if the illness continue long, assumes a dry brown appearance. There is not a very great prostration of the strength at the beginning, and the discharges are not always suppressed or altered. Although I do not at all wish to overrate the importance of the local affections, it cannot be denied that the most remarkable feature in the case is the extreme pain which the patient experiences in the swollen part; it is as acute in this as in any other disease which I have seen. The duration of the attack varies: one case terminated fatally within forty-eight hours, another was protracted to three weeks. The period also at which the patient is attacked after delivery is equally uncertain, varying from the second to the fourteenth day. I was inclined at first to consider the disease dependant on some inflammatory condition of the veins, originating probably in those of the womb, but examination of the veins of the pelvis, as well as of the veins of the affected part, has convinced me that this opinion is erroneous: and I regret to say that dissection has as yet thrown little or no light on this singular disease. The swelling of the limbs is occasioned by serous effusion into the cellular tissue, and we generally find a similar effusion into the cavity which suffers most during the attack. Dr. Lee, in his valuable paper on Inflammation of the Veins of the Uterus, alludes to some fatal cases of this kind in which the veins of the womb were perfectly healthy, but offers no opinion on the nature of the disease itself. When the joints are attacked in uterine phlebitis, there are generally purulent depositions with destruction of the cartilages, whereas, in the complaint to which I have been directing the attention of the Society, I have never seen depositions of pus, and the interior of the joints are never affected. The swelling (even when it extends along the whole of the limb) differs also from that which is observed in phlegmasiæ dolens: in the latter disease, there is no discoloration of the integuments, excepting in its advanced stage, and then black spots occasionally occur, no doubt from extravasation of blood. In the former, the discoloration exists almost from the commencement, and is of a dusky red colour, very similar to what is observed in gouty inflammation, and does not alter in the progress of the disease.

The different plans of treatment which have been hitherto adopted have been found equally unavailing. In the two first cases no local means were tried to the swollen part; in the subsequent cases copious local bleeding was resorted to, and, if with no other benefit, the severity of the pain was relieved. If the pulse is very full at the commencement, general bleeding was employed. The internal remedies have usually consisted of antimonial and diaphoretic medicines.

---

